# Everolimus-induced acute fibrinous and organizing pneumonia (AFOP)

**DOI:** 10.5339/qmj.2024.qitc.14

**Published:** 2024-03-25

**Authors:** Theeb Osama Sulaiman, Mona Al-Langawi, Mushtaq Ahmed, Abdullah Arshad

**Affiliations:** 1 Pulmonology Department, Hamad General Hospital, Hamad Medical Corporation, Doha, Qatar Email: tsulaiman@hamad.qa

**Keywords:** Everolimus, Acute fibrinous and organizing pneumonia, Steroid

## Introduction

A variety of conditions have been associated with acute fibrinous and organizing pneumonia (AFOP), including autoimmune disorders, infectious agents, post-transplantation complications, and drugs.^1,2^ We describe a patient with a history of breast cancer who developed AFOP secondary to everolimus, and the condition improved with discontinuation of the drug and steroid treatment.

## Case Presentation

**Figure 1.**
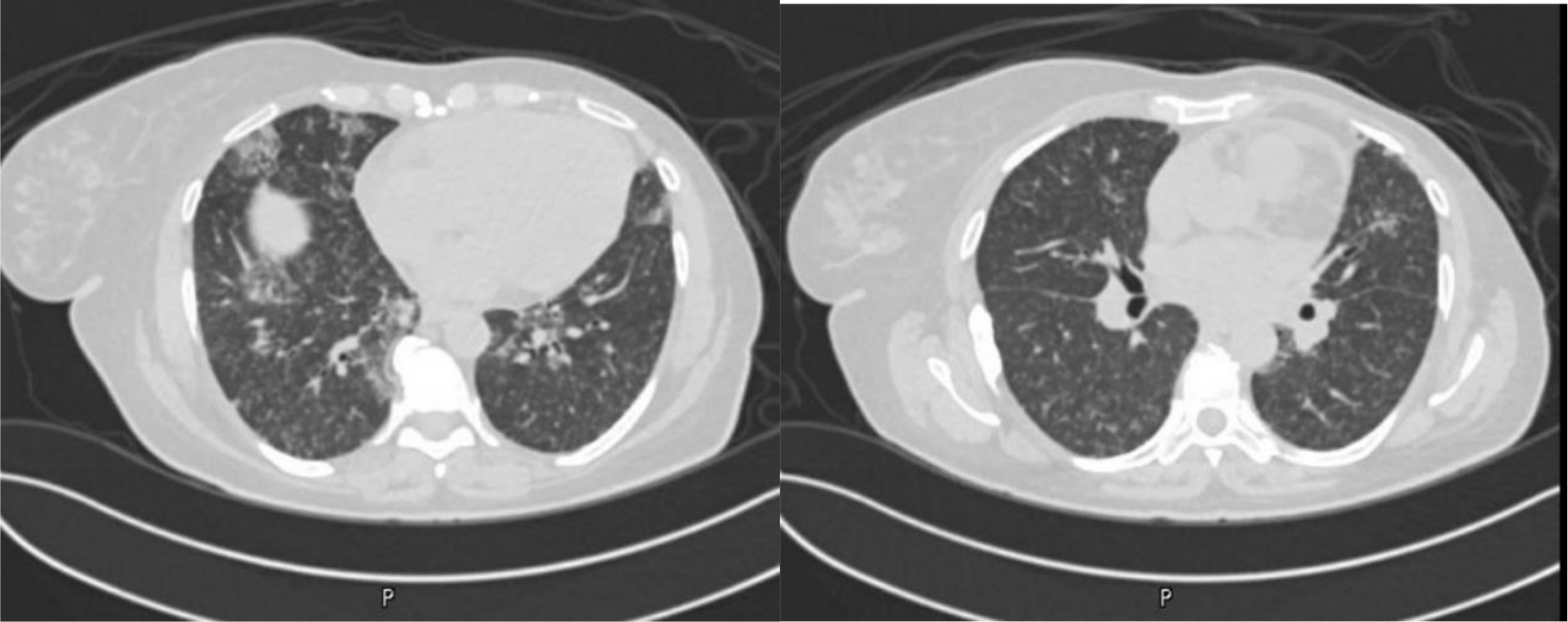
Chest CT scan showing diffuse numerous tiny nodules in both lung fields with areas of patchy GGOs and intralobular thickening more pronounced in both lower lobes.

**Figure 2.**
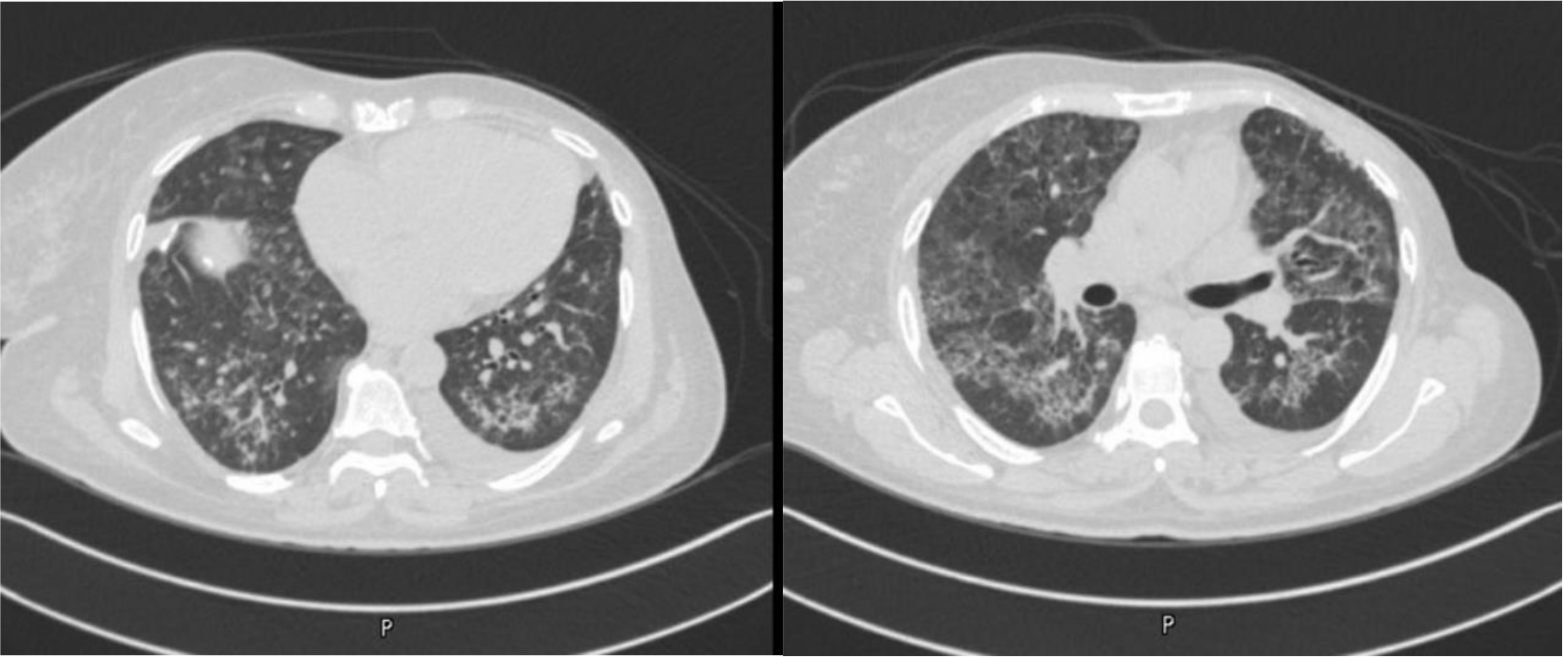
Chest CT scan showing progression of GGOs compared to Figure 1.

**Figure 3.**
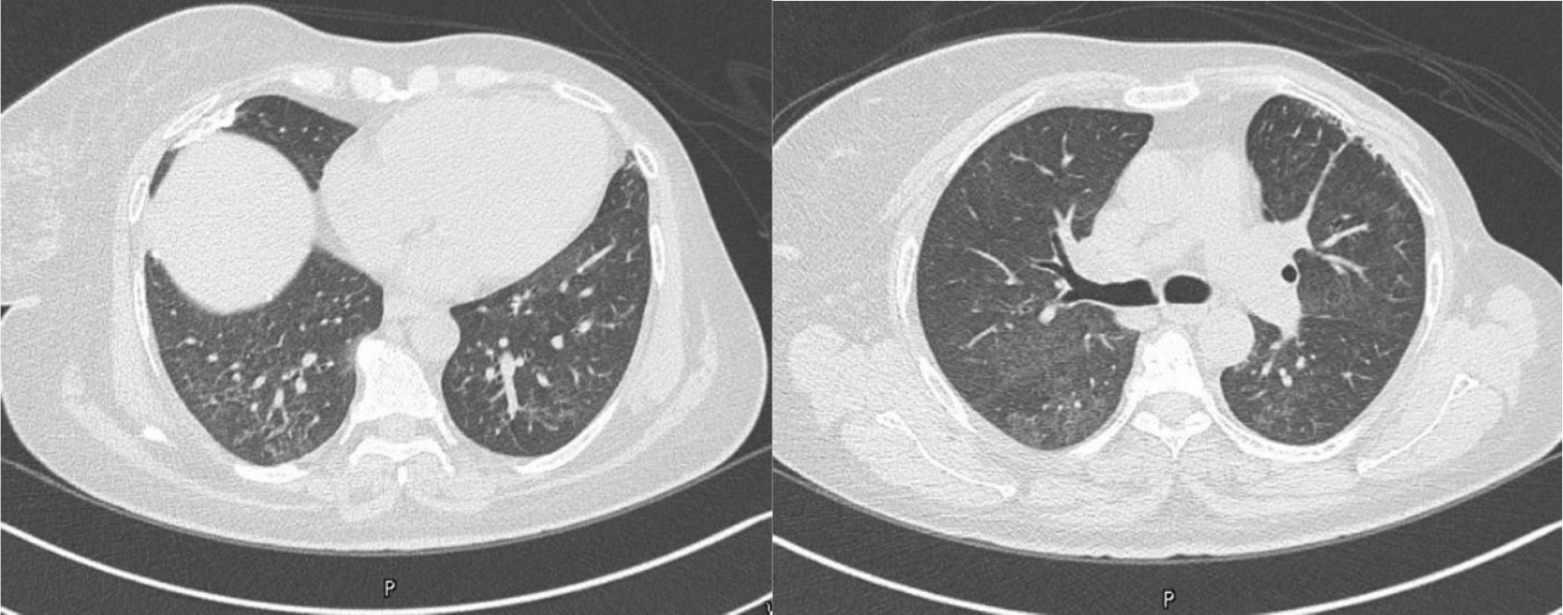
Chest CT scan showing appreciable regression of the previously reported bilateral consolidation, ground-glassing, and reticular patterns.

A 54-year-old female patient with a history of advanced breast cancer who was on everolimus was found to have diffuse tiny lung nodules, accompanied by patchy ground-glass opacities (GGOs), on routine surveillance imaging (Figure 1). At that time, she was asymptomatic. Bronchoalveolar lavage workup was unremarkable, and a transbronchial biopsy failed to provide a pathological diagnosis. Subsequently, a surgical lung biopsy was performed, and histopathology revealed findings consistent with AFOP. A few days later, she was admitted with dyspnea and a productive cough. Vital signs were stable, except for mild hypoxia, and chest examination showed basal decreased air entry with coarse crackles on the right side. Other physical examinations were unremarkable. Laboratory results showed an elevated white blood cell count, mildly elevated C-reactive protein, and an NT-pro-BNP level of 4,929 pg/mL, and echocardiography revealed an EF of 37%. The patient was managed with antibiotics and furosemide, but without significant improvement. A new HRCT showed worsening of the patchy pulmonary opacities (Figure 2). Everolimus was stopped, and the patient was started on prednisone 30 mg daily for 4 weeks, followed by a gradual tapering. Follow-up imaging after 2 months showed regression of previously reported findings with significant improvement in symptoms (Figure 3).

## Conclusion

The association between everolimus and AFOP is extremely rare and not fully understood. Several treatment options are available for the management of AFOP. In the case of drug-induced AFOP, treatment primarily includes discontinuation of the offending drug and administration of steroid therapy. However, there is no consensus on the optimal dosage, tapering regimen, and the duration.

## Conflict of Interest

I have no conflict of interest.
